# 2,2,4,4-Tetra­phenyl-1,3-bis­(3,3,5,5-tetra­methyl-1,1-diphenyl-5-vinyl­trisilox­an-1-yl)­cyclo­disilazane

**DOI:** 10.1107/S1600536811014863

**Published:** 2011-04-29

**Authors:** Zhen Lv, Lina Dai, Xuezhong Zhang, Zhijie Zhang, Zemin Xie

**Affiliations:** a Institute of Chemistry, Chinese Academy of Sciences, Beijing 100190, People’s Republic of China

## Abstract

The title mol­ecule, C_60_H_70_N_2_O_4_Si_8_, lies on an inversion center. In the asymmetric unit, one of the phenyl rings is disordered over two sets of sites with refined occupancies 0.58 (2) and 0.42 (2). In addition, in two substitution sites of the terminal dimeth­yl(vin­yl)silyl unit, a methyl group and the vinyl group are disordered over the same site with refined occupancies 0.523 (13) and 0.477 (13).

## Related literature

For similar cyclo­disilaza­nes to the title compound and their synthesis, see: Zhu *et al.* (2007 ▶).
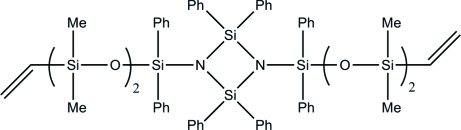


## Experimental

### 

#### Crystal data

C_60_H_70_N_2_O_4_Si_8_
*M* *_r_* = 1107.90
Triclinic, 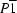

*a* = 10.731 (2) Å
*b* = 11.021 (2) Å
*c* = 13.859 (3) Å
α = 88.320 (9)°
β = 76.120 (6)°
γ = 78.860 (8)°
*V* = 1561.0 (6) Å^3^
*Z* = 1
Mo *K*α radiation
μ = 0.22 mm^−1^
*T* = 173 K
0.41 × 0.28 × 0.22 mm

#### Data collection

Rigaku Saturn724+ CCD diffractometer
Absorption correction: multi-scan (*CrystalClear*; Rigaku, 2007 ▶) *T*_min_ = 0.916, *T*_max_ = 0.954
12950 measured reflections
6338 independent reflections
5389 reflections with *I* > 2σ(*I*)
*R*_int_ = 0.040

#### Refinement

*R*[*F* ^2^ > 2σ(*F* ^2^)] = 0.072
*wR*(*F* ^2^) = 0.169
*S* = 1.11
6338 reflections
390 parameters
74 restraints
H-atom parameters constrained
Δρ_max_ = 0.55 e Å^−3^
Δρ_min_ = −0.32 e Å^−3^

### 

Data collection: *CrystalClear* (Rigaku, 2007 ▶); cell refinement: *CrystalClear*; data reduction: *CrystalClear*; program(s) used to solve structure: *SHELXS97* (Sheldrick, 2008 ▶); program(s) used to refine structure: *SHELXL97* (Sheldrick, 2008 ▶); molecular graphics: *SHELXTL* (Sheldrick, 2008 ▶); software used to prepare material for publication: *SHELXL97*.

## Supplementary Material

Crystal structure: contains datablocks I, global. DOI: 10.1107/S1600536811014863/lh5235sup1.cif

Structure factors: contains datablocks I. DOI: 10.1107/S1600536811014863/lh5235Isup2.hkl

Additional supplementary materials:  crystallographic information; 3D view; checkCIF report

## Figures and Tables

**Fig. 1.**
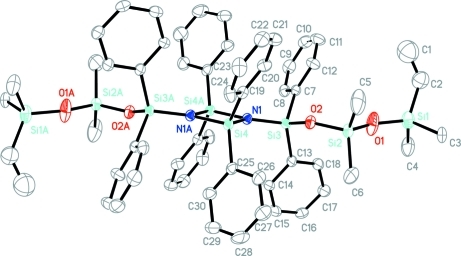
The molecular structure of the title compound shown with 30% ellipsoids. H atoms are not shown (symmetry code: (A) -x+1, -y+1, -z+1). The disorder is not shown.

**Fig. 2.**
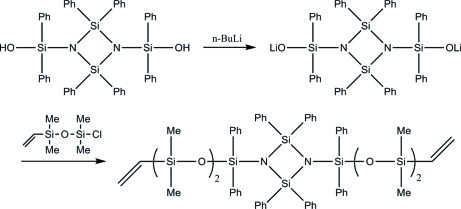
The synthesis of the title compound.

## supplementary crystallographic information

### Comment

Organocyclodisilazanes exhibit high thermal stability, and have been used to enhance the thermal properties of silicone rubbers (Zhu *et al.*, 2007). Different functional groups can be introduced to cyclodisilazanes to improve their reactivity.

The molecular structure of the title compound is shown in Fig. 1. The molecule lies on an inversion center. In the asymmetric unit, one of the phenyl rings is disordered over two sets of sites with refined occupancies 0.58 (2) and 0.42 (2). In addition, in the the terminal dimethyl(vinyl)silyl group one of the methyl groups and the vinyl group are disordered over their respective sites with refined occupancies 0.523 (13) and 0.477 (13).

### Experimental

The reaction scheme is shown in Fig. 2. 2 g 1,3-bis-(hydroxydiphenylsilanyl)-2,2,4,4-tetraphenylcyclodisilazane was dissolved in 20 ml tetrahydrofuran and added dropwisely to 2 ml n-butyllithum (2.5 mol/*L* solution in n-hexane) at 263K, then warmed to room temperature. An excessive amount of 1-vinyl-3-chloro-1,1,3,3-tetramethyldisiloxane was added to the mixture. After stirring for 30 min, solvents and unreacted disiloxane were removed under reduced pressure. The crude product was recrystallized from n-hexane to give colorless crystal.

### Refinement

All  H atoms were located in difference maps but were constrained in a riding-model approximation with *U*_iso_(H) = 1.2*U*_eq_(C_aryl_) or *U*_iso_(H) = 1.5*U*_eq_(C_methyl_). C—H distances were constrained to 0.95 and 0.98 Å for aryl and methyl H atoms respectively.

### Crystal data

**Table:**

C_60_H_70_N_2_O_4_Si_8_	*Z* = 1
*M**_r_* = 1107.90	*F*(000) = 588
Triclinic, *P*1	*D*_x_ = 1.179 Mg m^−^^3^
Hall symbol: -P 1	Mo *K*α radiation, λ = 0.71073 Å
*a* = 10.731 (2) Å	Cell parameters from 4640 reflections
*b* = 11.021 (2) Å	θ = 1.5–26.4°
*c* = 13.859 (3) Å	µ = 0.22 mm^−^^1^
α = 88.320 (9)°	*T* = 173 K
β = 76.120 (6)°	Block, colorless
γ = 78.860 (8)°	0.41 × 0.28 × 0.22 mm
*V* = 1561.0 (6) Å^3^	

### Data collection

**Table:**

Rigaku Saturn724+ CCD diffractometer	6338 independent reflections
Radiation source: sealed tube	5389 reflections with *I* > 2σ(*I*)
graphite	*R*_int_ = 0.040
ω scans at fixed χ = 45°	θ_max_ = 26.4°, θ_min_ = 2.4°
Absorption correction: multi-scan (*CrystalClear*; Rigaku, 2007)	*h* = −13→13
*T*_min_ = 0.916, *T*_max_ = 0.954	*k* = −13→13
12950 measured reflections	*l* = −17→17

### Refinement

**Table:**

Refinement on *F*^2^	Primary atom site location: structure-invariant direct methods
Least-squares matrix: full	Secondary atom site location: difference Fourier map
*R*[*F*^2^ > 2σ(*F*^2^)] = 0.072	Hydrogen site location: inferred from neighbouring sites
*wR*(*F*^2^) = 0.169	H-atom parameters constrained
*S* = 1.11	*w* = 1/[σ^2^(*F*_o_^2^) + (0.0572*P*)^2^ + 1.4799*P*] where *P* = (*F*_o_^2^ + 2*F*_c_^2^)/3
6338 reflections	(Δ/σ)_max_ = 0.001
390 parameters	Δρ_max_ = 0.55 e Å^−^^3^
74 restraints	Δρ_min_ = −0.32 e Å^−^^3^

### Special details

**Table:**

Geometry. All e.s.d.'s (except the e.s.d. in the dihedral angle between two l.s. planes) are estimated using the full covariance matrix. The cell e.s.d.'s are taken into account individually in the estimation of e.s.d.'s in distances, angles and torsion angles; correlations between e.s.d.'s in cell parameters are only used when they are defined by crystal symmetry. An approximate (isotropic) treatment of cell e.s.d.'s is used for estimating e.s.d.'s involving l.s. planes.
Refinement. Refinement of *F*^2^ against ALL reflections. The weighted *R*-factor *wR* and goodness of fit *S* are based on *F*^2^, conventional *R*-factors *R* are based on *F*, with *F* set to zero for negative *F*^2^. The threshold expression of *F*^2^ > σ(*F*^2^) is used only for calculating *R*-factors(gt) *etc*. and is not relevant to the choice of reflections for refinement. *R*-factors based on *F*^2^ are statistically about twice as large as those based on *F*, and *R*- factors based on ALL data will be even larger.

### Fractional atomic coordinates and isotropic or equivalent isotropic displacement parameters (Å^2^)

**Table:**

	*x*	*y*	*z*	*U*_iso_*/*U*_eq_	Occ. (<1)
Si1	0.25363 (13)	0.87059 (11)	0.07043 (8)	0.0648 (3)	
Si2	0.35367 (9)	0.85862 (8)	0.26534 (7)	0.0431 (2)	
Si3	0.44457 (8)	0.58120 (7)	0.30819 (6)	0.0318 (2)	
Si4	0.49207 (8)	0.61058 (7)	0.52203 (6)	0.0325 (2)	
O1	0.3102 (4)	0.8299 (3)	0.1668 (2)	0.0902 (11)	
O2	0.3845 (2)	0.72946 (19)	0.32207 (15)	0.0411 (5)	
N1	0.4736 (2)	0.5313 (2)	0.42053 (17)	0.0314 (5)	
C4	0.3722 (5)	0.7923 (5)	−0.0386 (3)	0.0848 (15)	
H4A	0.3830	0.7025	−0.0303	0.127*	
H4B	0.4565	0.8174	−0.0451	0.127*	
H4C	0.3402	0.8151	−0.0984	0.127*	
C5	0.2206 (6)	0.9597 (5)	0.3495 (4)	0.111 (2)	
H5A	0.1435	0.9209	0.3658	0.167*	
H5B	0.1995	1.0389	0.3175	0.167*	
H5C	0.2471	0.9737	0.4105	0.167*	
C6	0.5004 (6)	0.9265 (6)	0.2308 (5)	0.125 (3)	
H6A	0.5695	0.8703	0.1848	0.187*	
H6B	0.5300	0.9400	0.2906	0.187*	
H6C	0.4803	1.0058	0.1985	0.187*	
C7	0.3206 (3)	0.5015 (3)	0.2770 (2)	0.0361 (6)	
C8	0.3566 (3)	0.3802 (3)	0.2406 (2)	0.0452 (8)	
H8A	0.4461	0.3409	0.2266	0.054*	
C9	0.2644 (4)	0.3153 (4)	0.2242 (3)	0.0568 (9)	
H9A	0.2910	0.2322	0.2002	0.068*	
C10	0.1356 (4)	0.3712 (4)	0.2427 (3)	0.0636 (11)	
H10A	0.0727	0.3266	0.2315	0.076*	
C11	0.0964 (4)	0.4913 (4)	0.2774 (3)	0.0625 (11)	
H11A	0.0068	0.5301	0.2895	0.075*	
C12	0.1883 (3)	0.5556 (4)	0.2945 (3)	0.0500 (8)	
H12A	0.1604	0.6386	0.3188	0.060*	
C13	0.5940 (3)	0.5541 (3)	0.2047 (2)	0.0390 (7)	
C14	0.7229 (9)	0.5085 (12)	0.2155 (7)	0.046 (2)	0.58 (2)
H14	0.7364	0.4915	0.2802	0.055*	0.58 (2)
C15	0.8279 (8)	0.4885 (15)	0.1357 (6)	0.060 (3)	0.58 (2)
H15	0.9133	0.4631	0.1462	0.072*	0.58 (2)
C16	0.8121 (10)	0.5046 (13)	0.0405 (8)	0.054 (2)	0.58 (2)
H16	0.8851	0.4852	−0.0147	0.064*	0.58 (2)
C14'	0.7159 (15)	0.5652 (18)	0.2198 (11)	0.056 (4)	0.42 (2)
H14'	0.7247	0.5753	0.2856	0.067*	0.42 (2)
C15'	0.8242 (12)	0.562 (2)	0.1404 (9)	0.065 (4)	0.42 (2)
H15'	0.9082	0.5607	0.1520	0.078*	0.42 (2)
C16'	0.8082 (16)	0.5605 (18)	0.0437 (12)	0.061 (4)	0.42 (2)
H16'	0.8796	0.5671	−0.0107	0.073*	0.42 (2)
C17	0.6879 (4)	0.5495 (4)	0.0261 (3)	0.0604 (10)	
H17A	0.6763	0.5714	−0.0384	0.072*	
C18	0.5815 (3)	0.5621 (3)	0.1065 (2)	0.0489 (8)	
H18A	0.4963	0.5769	0.0947	0.059*	
C19	0.3401 (3)	0.7168 (3)	0.5883 (2)	0.0379 (7)	
C20	0.2205 (3)	0.7236 (3)	0.5635 (2)	0.0455 (8)	
H20A	0.2164	0.6758	0.5088	0.055*	
C21	0.1073 (4)	0.7988 (3)	0.6170 (3)	0.0542 (9)	
H21A	0.0269	0.8016	0.5988	0.065*	
C22	0.1108 (4)	0.8692 (3)	0.6962 (3)	0.0571 (10)	
H22A	0.0331	0.9202	0.7328	0.069*	
C23	0.2274 (4)	0.8653 (3)	0.7220 (3)	0.0596 (10)	
H23A	0.2301	0.9137	0.7768	0.072*	
C24	0.3415 (4)	0.7910 (3)	0.6683 (3)	0.0488 (8)	
H24A	0.4218	0.7906	0.6861	0.059*	
C25	0.6302 (3)	0.6964 (3)	0.4937 (2)	0.0396 (7)	
C26	0.6095 (4)	0.8199 (4)	0.4685 (3)	0.0632 (11)	
H26A	0.5233	0.8626	0.4695	0.076*	
C27	0.7126 (5)	0.8820 (4)	0.4419 (4)	0.0888 (16)	
H27A	0.6965	0.9666	0.4244	0.107*	
C28	0.8367 (5)	0.8235 (5)	0.4405 (4)	0.0767 (13)	
H28A	0.9069	0.8670	0.4216	0.092*	
C29	0.8607 (4)	0.7013 (4)	0.4664 (3)	0.0577 (10)	
H29A	0.9470	0.6603	0.4667	0.069*	
C30	0.7575 (3)	0.6387 (3)	0.4920 (2)	0.0475 (8)	
H30A	0.7745	0.5539	0.5088	0.057*	
C1	0.0010 (15)	0.8122 (14)	0.1453 (11)	0.143 (6)	0.523 (13)
H1A	0.0172	0.7954	0.2092	0.172*	0.523 (13)
H1B	−0.0805	0.8042	0.1331	0.172*	0.523 (13)
C2	0.0907 (15)	0.8463 (12)	0.0740 (10)	0.088 (4)	0.523 (13)
H2	0.0658	0.8610	0.0127	0.105*	0.523 (13)
C3	0.2438 (10)	1.0414 (7)	0.0555 (6)	0.047 (2)	0.523 (13)
H3A	0.1811	1.0851	0.1130	0.071*	0.523 (13)
H3B	0.2151	1.0672	−0.0053	0.071*	0.523 (13)
H3C	0.3302	1.0613	0.0509	0.071*	0.523 (13)
C1'	0.269 (2)	1.105 (2)	0.0102 (17)	0.202 (11)	0.477 (13)
H1'1	0.3555	1.0657	−0.0206	0.242*	0.477 (13)
H1'2	0.2415	1.1916	0.0029	0.242*	0.477 (13)
C2'	0.187 (2)	1.041 (2)	0.0620 (16)	0.147 (9)	0.477 (13)
H2'	0.0998	1.0778	0.0937	0.177*	0.477 (13)
C3'	0.1027 (19)	0.797 (2)	0.0976 (15)	0.122 (7)	0.477 (13)
H3'1	0.0388	0.8382	0.1558	0.183*	0.477 (13)
H3'2	0.1276	0.7090	0.1111	0.183*	0.477 (13)
H3'3	0.0638	0.8060	0.0400	0.183*	0.477 (13)

### Atomic displacement parameters (Å^2^)

**Table:**

	*U*^11^	*U*^22^	*U*^33^	*U*^12^	*U*^13^	*U*^23^
Si1	0.0854 (9)	0.0687 (7)	0.0447 (6)	−0.0075 (6)	−0.0297 (6)	0.0025 (5)
Si2	0.0505 (5)	0.0395 (5)	0.0392 (5)	−0.0033 (4)	−0.0152 (4)	0.0057 (4)
Si3	0.0321 (4)	0.0377 (4)	0.0253 (4)	−0.0043 (3)	−0.0083 (3)	0.0021 (3)
Si4	0.0369 (4)	0.0349 (4)	0.0267 (4)	−0.0082 (3)	−0.0087 (3)	0.0027 (3)
O1	0.164 (3)	0.0658 (19)	0.0599 (19)	−0.024 (2)	−0.064 (2)	0.0151 (15)
O2	0.0486 (13)	0.0378 (12)	0.0338 (11)	−0.0020 (9)	−0.0093 (9)	0.0046 (9)
N1	0.0355 (13)	0.0341 (13)	0.0264 (12)	−0.0079 (10)	−0.0098 (10)	0.0009 (9)
C4	0.109 (4)	0.082 (3)	0.053 (3)	0.007 (3)	−0.022 (3)	0.007 (2)
C5	0.144 (5)	0.077 (3)	0.071 (3)	0.053 (3)	−0.005 (3)	0.006 (3)
C6	0.106 (4)	0.127 (5)	0.170 (6)	−0.065 (4)	−0.064 (4)	0.101 (5)
C7	0.0369 (16)	0.0471 (18)	0.0243 (14)	−0.0078 (13)	−0.0077 (12)	0.0034 (12)
C8	0.051 (2)	0.052 (2)	0.0345 (16)	−0.0103 (15)	−0.0155 (14)	0.0023 (14)
C9	0.076 (3)	0.053 (2)	0.049 (2)	−0.0226 (19)	−0.0210 (19)	−0.0023 (16)
C10	0.068 (3)	0.089 (3)	0.050 (2)	−0.045 (2)	−0.0208 (19)	0.007 (2)
C11	0.039 (2)	0.094 (3)	0.058 (2)	−0.018 (2)	−0.0143 (17)	−0.003 (2)
C12	0.0349 (17)	0.062 (2)	0.053 (2)	−0.0062 (15)	−0.0121 (15)	−0.0067 (16)
C13	0.0381 (17)	0.0496 (18)	0.0278 (15)	−0.0054 (14)	−0.0076 (12)	0.0044 (13)
C14	0.032 (3)	0.067 (6)	0.037 (4)	−0.010 (4)	−0.009 (3)	0.011 (4)
C15	0.034 (3)	0.095 (7)	0.048 (4)	−0.011 (4)	−0.007 (3)	0.009 (4)
C16	0.040 (4)	0.071 (6)	0.042 (4)	−0.012 (4)	0.005 (3)	0.006 (5)
C14'	0.049 (5)	0.076 (8)	0.035 (5)	0.003 (6)	−0.010 (4)	−0.003 (6)
C15'	0.045 (5)	0.098 (9)	0.053 (5)	−0.019 (6)	−0.012 (4)	0.007 (6)
C16'	0.058 (6)	0.072 (8)	0.044 (5)	−0.008 (6)	−0.002 (4)	0.010 (6)
C17	0.050 (2)	0.101 (3)	0.0295 (17)	−0.017 (2)	−0.0073 (15)	0.0096 (18)
C18	0.0416 (18)	0.072 (2)	0.0311 (16)	−0.0078 (16)	−0.0085 (14)	0.0045 (15)
C19	0.0443 (17)	0.0364 (16)	0.0327 (15)	−0.0079 (13)	−0.0086 (13)	0.0032 (12)
C20	0.0467 (19)	0.0465 (19)	0.0401 (18)	−0.0099 (15)	−0.0024 (14)	−0.0070 (14)
C21	0.0421 (19)	0.060 (2)	0.055 (2)	−0.0064 (16)	−0.0035 (16)	−0.0028 (17)
C22	0.057 (2)	0.047 (2)	0.051 (2)	0.0069 (17)	0.0053 (17)	−0.0043 (16)
C23	0.078 (3)	0.047 (2)	0.045 (2)	0.0090 (18)	−0.0144 (19)	−0.0126 (16)
C24	0.060 (2)	0.0404 (18)	0.0448 (19)	0.0044 (15)	−0.0214 (16)	−0.0045 (14)
C25	0.0485 (18)	0.0440 (18)	0.0308 (15)	−0.0164 (14)	−0.0124 (13)	0.0038 (12)
C26	0.066 (3)	0.057 (2)	0.077 (3)	−0.0246 (19)	−0.029 (2)	0.024 (2)
C27	0.087 (4)	0.066 (3)	0.130 (5)	−0.043 (3)	−0.041 (3)	0.046 (3)
C28	0.073 (3)	0.082 (3)	0.089 (3)	−0.052 (3)	−0.019 (2)	0.017 (3)
C29	0.046 (2)	0.076 (3)	0.053 (2)	−0.0228 (18)	−0.0060 (16)	−0.0019 (19)
C30	0.0470 (19)	0.054 (2)	0.0413 (18)	−0.0131 (15)	−0.0065 (15)	−0.0004 (15)
C1	0.144 (10)	0.154 (10)	0.133 (9)	−0.024 (8)	−0.039 (8)	0.000 (7)
C2	0.099 (7)	0.080 (7)	0.089 (7)	−0.026 (6)	−0.026 (6)	0.003 (6)
C3	0.063 (5)	0.039 (4)	0.042 (4)	−0.001 (3)	−0.025 (3)	0.009 (3)
C1'	0.222 (14)	0.194 (13)	0.196 (14)	−0.045 (9)	−0.055 (9)	−0.013 (9)
C2'	0.146 (12)	0.171 (12)	0.129 (11)	−0.044 (9)	−0.027 (8)	−0.016 (8)
C3'	0.118 (10)	0.127 (11)	0.119 (10)	−0.028 (8)	−0.025 (8)	0.018 (8)

### Geometric parameters (Å, °)

**Table:**

Si1—O1	1.613 (3)	C16—C17	1.389 (12)
Si1—C2	1.808 (15)	C16—H16	0.9500
Si1—C4	1.837 (5)	C14'—C15'	1.392 (19)
Si1—C3	1.874 (8)	C14'—H14'	0.9500
Si1—C2'	1.89 (2)	C15'—C16'	1.39 (2)
Si1—C3'	1.90 (2)	C15'—H15'	0.9500
Si2—O1	1.603 (3)	C16'—C17	1.398 (18)
Si2—O2	1.622 (2)	C16'—H16'	0.9500
Si2—C5	1.817 (5)	C17—C18	1.379 (5)
Si2—C6	1.828 (5)	C17—H17A	0.9500
Si3—O2	1.635 (2)	C18—H18A	0.9500
Si3—N1	1.715 (2)	C19—C20	1.394 (5)
Si3—C13	1.859 (3)	C19—C24	1.402 (4)
Si3—C7	1.865 (3)	C20—C21	1.388 (5)
Si4—N1	1.748 (2)	C20—H20A	0.9500
Si4—N1^i^	1.749 (2)	C21—C22	1.374 (5)
Si4—C19	1.865 (3)	C21—H21A	0.9500
Si4—C25	1.867 (3)	C22—C23	1.374 (6)
Si4—Si4^i^	2.4940 (17)	C22—H22A	0.9500
N1—Si4^i^	1.749 (2)	C23—C24	1.391 (5)
C4—H4A	0.9800	C23—H23A	0.9500
C4—H4B	0.9800	C24—H24A	0.9500
C4—H4C	0.9800	C25—C26	1.385 (5)
C5—H5A	0.9800	C25—C30	1.386 (5)
C5—H5B	0.9800	C26—C27	1.382 (6)
C5—H5C	0.9800	C26—H26A	0.9500
C6—H6A	0.9800	C27—C28	1.360 (6)
C6—H6B	0.9800	C27—H27A	0.9500
C6—H6C	0.9800	C28—C29	1.375 (6)
C7—C8	1.393 (4)	C28—H28A	0.9500
C7—C12	1.395 (4)	C29—C30	1.386 (5)
C8—C9	1.390 (5)	C29—H29A	0.9500
C8—H8A	0.9500	C30—H30A	0.9500
C9—C10	1.367 (6)	C1—C2	1.306 (9)
C9—H9A	0.9500	C1—H1A	0.9500
C10—C11	1.373 (6)	C1—H1B	0.9500
C10—H10A	0.9500	C2—H2	0.9500
C11—C12	1.388 (5)	C3—H3A	0.9800
C11—H11A	0.9500	C3—H3B	0.9800
C12—H12A	0.9500	C3—H3C	0.9800
C13—C18	1.397 (4)	C1'—C2'	1.298 (10)
C13—C14'	1.402 (17)	C1'—H1'1	0.9500
C13—C14	1.419 (11)	C1'—H1'2	0.9500
C14—C15	1.365 (12)	C2'—H2'	0.9500
C14—H14	0.9500	C3'—H3'1	0.9800
C15—C16	1.372 (14)	C3'—H3'2	0.9800
C15—H15	0.9500	C3'—H3'3	0.9800
			
O1—Si1—C2	116.9 (5)	C13—C14—H14	119.1
O1—Si1—C4	107.6 (2)	C14—C15—C16	121.1 (8)
C2—Si1—C4	110.8 (5)	C14—C15—H15	119.5
O1—Si1—C3	108.2 (3)	C16—C15—H15	119.5
C2—Si1—C3	104.9 (5)	C15—C16—C17	119.0 (8)
C4—Si1—C3	108.1 (3)	C15—C16—H16	120.5
O1—Si1—C2'	115.7 (7)	C17—C16—H16	120.5
C2—Si1—C2'	86.9 (8)	C15'—C14'—C13	121.2 (12)
C4—Si1—C2'	118.0 (7)	C15'—C14'—H14'	119.4
C3—Si1—C2'	18.1 (7)	C13—C14'—H14'	119.4
O1—Si1—C3'	100.8 (6)	C14'—C15'—C16'	119.3 (13)
C2—Si1—C3'	19.4 (7)	C14'—C15'—H15'	120.4
C4—Si1—C3'	108.9 (7)	C16'—C15'—H15'	120.4
C3—Si1—C3'	122.4 (7)	C15'—C16'—C17	120.5 (12)
C2'—Si1—C3'	104.3 (8)	C15'—C16'—H16'	119.7
O1—Si2—O2	108.31 (14)	C17—C16'—H16'	119.7
O1—Si2—C5	110.3 (2)	C18—C17—C16	119.3 (5)
O2—Si2—C5	107.30 (19)	C18—C17—C16'	117.6 (7)
O1—Si2—C6	109.3 (3)	C16—C17—C16'	25.4 (6)
O2—Si2—C6	109.92 (19)	C18—C17—H17A	120.4
C5—Si2—C6	111.7 (3)	C16—C17—H17A	120.4
O2—Si3—N1	106.34 (11)	C16'—C17—H17A	115.8
O2—Si3—C13	109.79 (13)	C17—C18—C13	122.6 (3)
N1—Si3—C13	113.21 (13)	C17—C18—H18A	118.7
O2—Si3—C7	108.97 (13)	C13—C18—H18A	118.7
N1—Si3—C7	109.71 (12)	C20—C19—C24	117.1 (3)
C13—Si3—C7	108.74 (14)	C20—C19—Si4	122.4 (2)
N1—Si4—N1^i^	89.00 (11)	C24—C19—Si4	120.5 (2)
N1—Si4—C19	114.02 (13)	C21—C20—C19	121.4 (3)
N1^i^—Si4—C19	114.79 (12)	C21—C20—H20A	119.3
N1—Si4—C25	114.72 (13)	C19—C20—H20A	119.3
N1^i^—Si4—C25	114.17 (13)	C22—C21—C20	120.4 (4)
C19—Si4—C25	109.14 (14)	C22—C21—H21A	119.8
N1—Si4—Si4^i^	44.52 (8)	C20—C21—H21A	119.8
N1^i^—Si4—Si4^i^	44.48 (8)	C23—C22—C21	119.6 (3)
C19—Si4—Si4^i^	125.40 (11)	C23—C22—H22A	120.2
C25—Si4—Si4^i^	125.46 (11)	C21—C22—H22A	120.2
Si2—O1—Si1	153.0 (2)	C22—C23—C24	120.4 (3)
Si2—O2—Si3	145.38 (14)	C22—C23—H23A	119.8
Si3—N1—Si4	131.82 (15)	C24—C23—H23A	119.8
Si3—N1—Si4^i^	137.03 (14)	C23—C24—C19	121.1 (3)
Si4—N1—Si4^i^	91.00 (11)	C23—C24—H24A	119.5
Si1—C4—H4A	109.5	C19—C24—H24A	119.5
Si1—C4—H4B	109.5	C26—C25—C30	117.3 (3)
H4A—C4—H4B	109.5	C26—C25—Si4	120.6 (3)
Si1—C4—H4C	109.5	C30—C25—Si4	122.0 (2)
H4A—C4—H4C	109.5	C27—C26—C25	120.9 (4)
H4B—C4—H4C	109.5	C27—C26—H26A	119.6
Si2—C5—H5A	109.5	C25—C26—H26A	119.6
Si2—C5—H5B	109.5	C28—C27—C26	120.8 (4)
H5A—C5—H5B	109.5	C28—C27—H27A	119.6
Si2—C5—H5C	109.5	C26—C27—H27A	119.6
H5A—C5—H5C	109.5	C27—C28—C29	120.0 (4)
H5B—C5—H5C	109.5	C27—C28—H28A	120.0
Si2—C6—H6A	109.5	C29—C28—H28A	120.0
Si2—C6—H6B	109.5	C28—C29—C30	119.2 (4)
H6A—C6—H6B	109.5	C28—C29—H29A	120.4
Si2—C6—H6C	109.5	C30—C29—H29A	120.4
H6A—C6—H6C	109.5	C29—C30—C25	121.8 (3)
H6B—C6—H6C	109.5	C29—C30—H30A	119.1
C8—C7—C12	117.0 (3)	C25—C30—H30A	119.1
C8—C7—Si3	120.2 (2)	C2—C1—H1A	120.0
C12—C7—Si3	122.7 (2)	C2—C1—H1B	120.0
C9—C8—C7	121.4 (3)	H1A—C1—H1B	120.0
C9—C8—H8A	119.3	C1—C2—Si1	131.9 (13)
C7—C8—H8A	119.3	C1—C2—H2	114.1
C10—C9—C8	119.9 (4)	Si1—C2—H2	114.1
C10—C9—H9A	120.0	Si1—C3—H3A	109.5
C8—C9—H9A	120.0	Si1—C3—H3B	109.5
C9—C10—C11	120.4 (3)	H3A—C3—H3B	109.5
C9—C10—H10A	119.8	Si1—C3—H3C	109.5
C11—C10—H10A	119.8	H3A—C3—H3C	109.5
C10—C11—C12	119.6 (4)	H3B—C3—H3C	109.5
C10—C11—H11A	120.2	C2'—C1'—H1'1	120.0
C12—C11—H11A	120.2	C2'—C1'—H1'2	120.0
C11—C12—C7	121.6 (4)	H1'1—C1'—H1'2	120.0
C11—C12—H12A	119.2	C1'—C2'—Si1	116 (2)
C7—C12—H12A	119.2	C1'—C2'—H2'	122.1
C18—C13—C14'	116.4 (7)	Si1—C2'—H2'	122.1
C18—C13—C14	114.9 (5)	Si1—C3'—H3'1	109.5
C14'—C13—C14	25.3 (6)	Si1—C3'—H3'2	109.5
C18—C13—Si3	119.4 (2)	H3'1—C3'—H3'2	109.5
C14'—C13—Si3	121.1 (6)	Si1—C3'—H3'3	109.5
C14—C13—Si3	125.1 (4)	H3'1—C3'—H3'3	109.5
C15—C14—C13	121.9 (8)	H3'2—C3'—H3'3	109.5
C15—C14—H14	119.1		
			
O2—Si2—O1—Si1	171.8 (6)	C14—C13—C14'—C15'	82 (2)
C5—Si2—O1—Si1	54.7 (7)	Si3—C13—C14'—C15'	−171.0 (9)
C6—Si2—O1—Si1	−68.4 (7)	C13—C14'—C15'—C16'	6.8 (19)
C2—Si1—O1—Si2	−108.4 (8)	C14'—C15'—C16'—C17	−7(2)
C4—Si1—O1—Si2	126.1 (6)	C15—C16—C17—C18	−8.1 (12)
C3—Si1—O1—Si2	9.6 (8)	C15—C16—C17—C16'	85 (2)
C2'—Si1—O1—Si2	−8.2 (10)	C15'—C16'—C17—C18	11.2 (15)
C3'—Si1—O1—Si2	−119.9 (9)	C15'—C16'—C17—C16	−89 (2)
O1—Si2—O2—Si3	36.2 (3)	C16—C17—C18—C13	12.4 (9)
C5—Si2—O2—Si3	155.2 (3)	C16'—C17—C18—C13	−16.5 (11)
C6—Si2—O2—Si3	−83.2 (4)	C14'—C13—C18—C17	16.4 (10)
N1—Si3—O2—Si2	163.1 (2)	C14—C13—C18—C17	−11.7 (8)
C13—Si3—O2—Si2	40.3 (3)	Si3—C13—C18—C17	176.5 (3)
C7—Si3—O2—Si2	−78.7 (3)	N1—Si4—C19—C20	5.1 (3)
O2—Si3—N1—Si4	−19.6 (2)	N1^i^—Si4—C19—C20	−95.6 (3)
C13—Si3—N1—Si4	101.0 (2)	C25—Si4—C19—C20	134.8 (3)
C7—Si3—N1—Si4	−137.33 (19)	Si4^i^—Si4—C19—C20	−45.1 (3)
O2—Si3—N1—Si4^i^	166.09 (19)	N1—Si4—C19—C24	−176.9 (2)
C13—Si3—N1—Si4^i^	−73.3 (2)	N1^i^—Si4—C19—C24	82.4 (3)
C7—Si3—N1—Si4^i^	48.4 (2)	C25—Si4—C19—C24	−47.2 (3)
N1^i^—Si4—N1—Si3	−176.1 (3)	Si4^i^—Si4—C19—C24	132.9 (2)
C19—Si4—N1—Si3	67.1 (2)	C24—C19—C20—C21	−1.3 (5)
C25—Si4—N1—Si3	−59.8 (2)	Si4—C19—C20—C21	176.8 (3)
Si4^i^—Si4—N1—Si3	−176.1 (3)	C19—C20—C21—C22	0.3 (6)
N1^i^—Si4—N1—Si4^i^	0.0	C20—C21—C22—C23	0.3 (6)
C19—Si4—N1—Si4^i^	−116.82 (13)	C21—C22—C23—C24	0.2 (6)
C25—Si4—N1—Si4^i^	116.28 (14)	C22—C23—C24—C19	−1.3 (6)
O2—Si3—C7—C8	166.1 (2)	C20—C19—C24—C23	1.8 (5)
N1—Si3—C7—C8	−77.9 (3)	Si4—C19—C24—C23	−176.3 (3)
C13—Si3—C7—C8	46.4 (3)	N1—Si4—C25—C26	93.3 (3)
O2—Si3—C7—C12	−18.2 (3)	N1^i^—Si4—C25—C26	−166.0 (3)
N1—Si3—C7—C12	97.9 (3)	C19—Si4—C25—C26	−36.0 (3)
C13—Si3—C7—C12	−137.8 (3)	Si4^i^—Si4—C25—C26	143.9 (3)
C12—C7—C8—C9	−1.2 (5)	N1—Si4—C25—C30	−83.4 (3)
Si3—C7—C8—C9	174.8 (3)	N1^i^—Si4—C25—C30	17.3 (3)
C7—C8—C9—C10	0.9 (5)	C19—Si4—C25—C30	147.3 (3)
C8—C9—C10—C11	0.0 (6)	Si4^i^—Si4—C25—C30	−32.9 (3)
C9—C10—C11—C12	−0.6 (6)	C30—C25—C26—C27	0.6 (6)
C10—C11—C12—C7	0.3 (6)	Si4—C25—C26—C27	−176.3 (4)
C8—C7—C12—C11	0.6 (5)	C25—C26—C27—C28	−0.5 (8)
Si3—C7—C12—C11	−175.3 (3)	C26—C27—C28—C29	−0.4 (8)
O2—Si3—C13—C18	−76.5 (3)	C27—C28—C29—C30	1.1 (7)
N1—Si3—C13—C18	164.8 (3)	C28—C29—C30—C25	−1.0 (6)
C7—Si3—C13—C18	42.6 (3)	C26—C25—C30—C29	0.1 (5)
O2—Si3—C13—C14'	82.7 (10)	Si4—C25—C30—C29	176.9 (3)
N1—Si3—C13—C14'	−36.0 (10)	O1—Si1—C2—C1	8.0 (18)
C7—Si3—C13—C14'	−158.2 (10)	C4—Si1—C2—C1	131.8 (15)
O2—Si3—C13—C14	112.6 (7)	C3—Si1—C2—C1	−111.8 (16)
N1—Si3—C13—C14	−6.0 (7)	C2'—Si1—C2—C1	−109.3 (17)
C7—Si3—C13—C14	−128.3 (7)	C3'—Si1—C2—C1	44 (3)
C18—C13—C14—C15	7.4 (10)	O1—Si1—C2'—C1'	94.4 (19)
C14'—C13—C14—C15	−92 (2)	C2—Si1—C2'—C1'	−147 (2)
Si3—C13—C14—C15	178.7 (7)	C4—Si1—C2'—C1'	−35 (2)
C13—C14—C15—C16	−4.1 (14)	C3—Si1—C2'—C1'	25.3 (18)
C14—C15—C16—C17	4.2 (14)	C3'—Si1—C2'—C1'	−155.9 (19)
C18—C13—C14'—C15'	−11.2 (15)		

Symmetry codes: (i) −*x*+1, −*y*+1, −*z*+1.
